# Long-Term, Real-World Kidney Outcomes with SGLT2i versus DPP4i in Type 2 Diabetes without Cardiovascular or Kidney Disease

**DOI:** 10.2215/CJN.0000000000000218

**Published:** 2023-06-29

**Authors:** Cheli Melzer Cohen, Meir Schechter, Aliza Rozenberg, Ilan Yanuv, Dvora R. Sehtman-Shachar, Alisa Fishkin, Doron Rosenzweig, Gabriel Chodick, Avraham Karasik, Ofri Mosenzon

**Affiliations:** 1 Maccabi Institute for Research and Innovation, Maccabi Healthcare Services, Tel-Aviv, Israel; 2 Diabetes Unit, Department of Endocrinology and Metabolism, Hadassah Medical Center, Jerusalem, Israel; 3 Faculty of Medicine, Hebrew University of Jerusalem, Jerusalem, Israel; 4 Department of Clinical Pharmacy and Pharmacology, University Medical Center Groningen, University of Groningen, Groningen, The Netherlands; 5 Boehringer Ingelheim RCV GmbH and Co. KG, Vienna, Austria; 6 School of Public Health Sackler, Faculty of Medicine, Tel Aviv University, Tel Aviv, Israel; 7 Tel Aviv University, Tel Aviv, Israel

**Keywords:** CKD, diabetes mellitus, SGLT2

## Abstract

**Background:**

Contemporary guidelines recommend the use of sodium-glucose cotransporter 2 inhibitors (SGLT2is) independently of glycemic control in patients with type 2 diabetes and those with kidney disease, with heart failure, or at high risk of cardiovascular disease. Using a large Israeli database, we assessed whether long-term use of SGLT2is versus dipeptidyl peptidase 4 inhibitors (DPP4is) is associated with kidney benefits in patients with type 2 diabetes overall and in those without evidence of cardiovascular or kidney disease.

**Methods:**

Patients with type 2 diabetes who initiated SGLT2is or DPP4is between 2015 and 2021 were propensity score-matched (1:1) according to 90 parameters. The kidney-specific composite outcome included confirmed ≥40% decline in eGFR or kidney failure. The kidney-or-death outcome included also all-cause mortality. Risks of outcomes were assessed using Cox proportional hazard regression models. The between-group difference in eGFR slope was also assessed. Analyses were repeated in patients' subgroup lacking evidence of cardiovascular or kidney disease.

**Results:**

Overall, 19,648 propensity score-matched patients were included; 10,467 (53%) did not have evidence of cardiovascular or kidney disease. Median follow-up was 38 months (interquartile range, 22–55). The composite kidney-specific outcome occurred at an event rate of 6.9 versus 9.5 events per 1000 patient-years with SGLT2i versus DPP4i. The respective event rates of the kidney-or-death outcome were 17.7 versus 22.1. Compared with DPP4is, initiation of SGLT2is was associated with a lower risk for the kidney-specific (hazard ratio [HR], 0.72; 95% confidence interval [CI], 0.61 to 0.86; *P* < 0.001) and kidney-or-death (HR, 0.80; 95% CI, 0.71 to 0.89; *P* < 0.001) outcomes. The respective HRs (95% CI) in those lacking evidence of cardiovascular or kidney disease were 0.67 (0.44 to 1.02) and 0.77 (0.61 to 0.97). Initiation of SGLT2is versus DPP4is was associated with mitigation of the eGFR slope overall and in those lacking evidence of cardiovascular or kidney disease (mean between-group differences 0.49 [95% CI, 0.35 to 0.62] and 0.48 [95% CI, 0.32 to 0.64] ml/min per 1.73 m^2^ per year, respectively).

**Conclusions:**

Long-term use of SGLT2is versus DPP4is in a real-world setting was associated with mitigation of eGFR loss in patients with type 2 diabetes, even in those lacking evidence of cardiovascular or kidney disease at baseline.

## Visual Abstract

**Figure:**
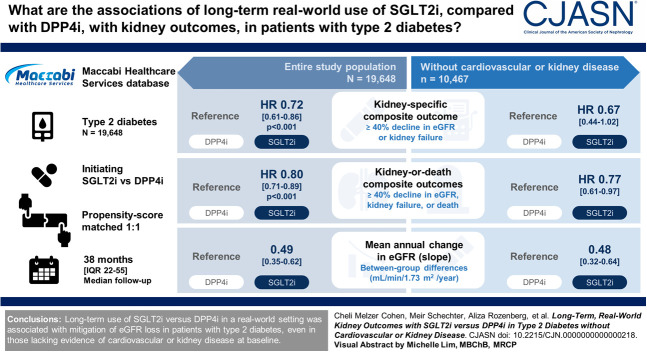

## Introduction

CKD is a common complication of type 2 diabetes, affecting approximately 20%–50% of the patients.^1–3^ It is commonly defined by a persistent (>3 months) decrease in eGFR, the presence of albuminuria, or other indications of kidney damage.^4^

Clinical trials in patients with type 2 diabetes at high cardiovascular or kidney risk showed that sodium-glucose cotransporter 2 inhibitors (SGLT2is) improve kidney outcomes.^5^ On the basis of these studies, current guidelines, including a recent consensus report by the American Diabetes Association and the Kidney Disease Improving Global Outcomes, recommend the use of SGLT2is independent of glycemic control in patients with type 2 diabetes and those with kidney disease, with heart failure, or at high risk of cardiovascular disease.^6,7^ Real-world studies further assessed the kidney effects of SGLT2is in broader populations of patients with type 2 diabetes that usually have a lower cardiovascular and kidney risk profile than the trial participants. For example, the Kidney Outcomes Associated with Use of SGLT2 Inhibitors in Real-World Clinical Practice (CVD-REAL 3) study demonstrated that initiation of SGLT2is is associated with a lower risk of kidney outcomes and mitigation of eGFR loss in a large, multinational real-world cohort.^8^ However, this study had a relatively short follow-up (mean 15 months), and the comparators were diverse and included any other glucose-lowering agent. We aimed to test the long-term association between use of SGLT2is, specifically empagliflozin, compared with dipeptidyl peptidase 4 inhibitors (DPP4is), and kidney outcomes, with specific emphasis on populations without evidence of cardiovascular and kidney disease.

## Methods

### Study Design, Participants, and Follow-Up Definitions

This study was conducted using the Maccabi Healthcare System (MHS) database, which has over 2 million participants with <1% yearly abandon rate. We included adults with type 2 diabetes^2^ who initiated any of the available SGLT2is in Israel (empagliflozin or dapagliflozin) or any available DPP4is (sitagliptin, linagliptin, vildagliptin, and saxagliptin) between August 2015 and December 2020. In addition, we compared initiators of empagliflozin with any of the available DPP4is. The day of drug dispensation was defined as the index date, and the preceding year was defined as the baseline period. We included patients with at least one eGFR measurement in the baseline period. We excluded patients with type 1 diabetes, eGFR <30 ml/min per 1.73 m^2^, history of kidney transplantation or dialysis treatment, or those using the comparator drug within the baseline period.

The study was approved by the institutional review board at MHS. Patients' informed consent was not needed because of the deidentified nature of the data.

### Definitions of Baseline Variables

Baseline variables were considered as the last measurement within the baseline period. Laboratory and clinical measurements were collected in community settings, and samples were analyzed in the MHS’s certified central laboratory. Creatinine was determined using the Jaffe method, and eGFR was calculated using the Chronic Kidney Disease Epidemiology Collaboration equation.^9^ Data of the residential socioeconomic status was obtained from the Israeli Central Bureau of Statistics as previously described.^10^ The International Classification of Diseases-9 (diagnoses), Anatomical Therapeutic Chemical medications codes, and MHS registries that we used in this study are presented in Supplemental Table 1A.

### Follow-Up Definitions

We used intention-to-treat and as-treated follow-up definitions. In the intention-to-treat definition, follow-up continued until the end of data availability, death, or September 2021. The as-treated definition's follow-up criteria were those used for the intention-to-treat definition, added by censoring at study drug discontinuation or the initiation of the comparator drug. In the as-treated definition, follow-up was extended by a grace period of 90 days after the last drug dispensation.

### Outcomes and Subgroup Definitions

The study had two main composite outcomes: a kidney-specific outcome composed of confirmed (two consecutive tests) ≥40% reduction from baseline eGFR or new kidney failure and a kidney-or-death outcome that included the components of the kidney-specific outcome or all-cause death. Additional outcomes were confirmed- or single-measurement eGFR reduction of ≥30%, ≥40%, ≥50%, or ≥57% (corresponding to a doubling of serum creatinine), new kidney failure, or all-cause death. We also assessed the risk of a categorical increase in the urine albumin-to-creatinine ratio (UACR) among patients with baseline UACR <300 for the following categories: <30, 30–<300, and ≥300 mg/g. In addition, we assessed the eGFR slopes during follow-up. Safety outcomes were not assessed in this study.

The outcomes were assessed in the whole study population and by the following subgroups: age (younger than 60 years or 60 years and older), sex, years in a diabetes registry (≤10 or >10), presence of cardiovascular disease (defined in the Supplemental Methods and Supplemental Table 1B), HbA1c (<8 or ≥8%), body mass index (<30 or ≥30 kg/m^2^), eGFR (≥90, 60–<90, or <60 ml/min per 1.73 m^2^), urine albumin (urine albumin less than detectable levels, UACR (<30, 30–<300, or ≥300 mg/g), and use of angiotensin-converting enzymes or angiotensin II receptor blockers. We specifically focused on a subgroup of patients with low cardiorenal risk defined by lack of all the following: evidence of cardiovascular disease, eGFR <60 ml/min per 1.73 m^2^, or UACR ≥30 mg/g.

### Statistical Analyses

Participants were propensity score-matched in a 1:1 ratio by layers of baseline eGFR (>90, 60–90, and <60 ml/min per 1.73 m^2^). Matching was performed using greedy matching, as previously described.^11^ The model included 90 baseline parameters (see the full list of demographics variables, medical history, concomitant medications, and laboratory values in the Supplemental Methods). For the matching process, missing values for continuous variables were imputed by using the mean value per study group. For categorical variables, missing values were classified into a missing category to allow all patients to be matched.

Continuous variables with approximately normal distribution were described as mean and SD, and those with skewed distribution as median and interquartile range (IQR). Categorical variables were described by proportions. Standardized difference was used to assess the baseline differences between the SGLT2i and DPP4i groups, with values >10% considered significant.

Incidence of the categorical outcomes was described using cumulative incidence functions. Cox proportional hazard regression models were applied to estimate hazard ratios (HRs), confidence intervals (CIs), and *P* values between the treatment arms. Competing risk of death was adjusted in the models using subdistribution hazard function and by using a cause-specific hazard model for cumulative incidence function.^12^ To test for heterogeneity by subgroups, the Cox models were adjusted to an interaction term of the treatment arm with the baseline subgroups.

Mixed models for repeated measures were used to describe the change in eGFR over time. We defined time windows as follows: every 3 months in the first year, every 6 months between years 1 and 3, and each year thereafter. At each time window, we considered only the eGFR measurement closest to the end of the period for each patient. We calculated the *P* value of the change in eGFR between groups at each time point, using a mixed-effect model with repeated measures. To compare the change in eGFR over time between the treatment groups using all available eGFR measurements per patient, we calculated an eGFR slope per patient by fitting a linear regression model. We then calculated the mean eGFR slope over time for each group and used a *t*-test to compare the treatment groups. In this analysis, we did not consider measurements that occurred within 28 days from the index date, to avoid over-representations of the reversible acute eGFR dip on the overall slope estimation.^13^

Analyses were performed using SAS version 9.4. The analyses did not include formal hypothesis testing, and the *P* values are presented for descriptive purposes, considering a *P* value of < 0.05 as statistically significant. No correction for multiple testing was performed.

### Role of the Funding Sources

This is an investigator-initiated analysis, and the study was funded by Boehringer Ingelheim. The investigators independently designed the study, analyzed the data, interpreted the findings, and wrote the report. The funder had the right to comment on the manuscript drafts.

## Results

### Baseline Characteristics

Between August 2015 and December 2020, 16,065 and 23,208 patients initiated SGLT2is and DPP4is, respectively (Supplemental Table 2). After propensity score-matching, there were 19,648 patients included in the analysis, of whom 7688 (39%) were women and 15,050 (77%) did not have evidence of cardiovascular disease. The mean age was 61 years (SD 12); the mean eGFR was 90 ml/min per 1.73 m^2^ (19); and the median UACR was 12 mg/g (IQR, 0–43). Overall, 10,467 patients (53%) had no evidence of cardiovascular or kidney disease (Table 1). Most (79%) of the participants in the SGLT2i arm initiated empagliflozin, and the rest initiated dapagliflozin. Baseline characteristics after propensity score-matching were balanced between the cohorts (with a standardized difference <10%) (Table 1).

**Table 1:** Baseline characteristics of patients initiating any sodium-glucose cotransporter 2 inhibitor or any dipeptidyl peptidase-4 inhibitor in the Maccabi Healthcare System, after propensity score-matching

Variable		SGLT2is (*n*=9824)	DPP4is (*n*=9824)
**Demographics**			
Age, yr, mean (SD)		61 (11)	61 (12)
Women, *n* (%)		3820 (39)	3868 (39)
SES,^a^ *n* (%)	1–3	1227 (13)	1190 (12)
	4–5	2911 (30)	2902 (30)
	6–7	3442 (35)	3466 (35)
	8–10	2229 (23)	2246 (23)
	Missing	15 (0.2)	20 (0.2)
Year of study entry, *n* (%)	2015–2016	2129 (22)	2262 (23)
	2017–2018	3723 (38)	3245 (33)
	2019–2020	3972 (40)	4344 (44)
**Medical history**			
Years in diabetes registry, mean (SD)		8 (6)	8 (6)
Established cardiovascular disease history,^b^ *n* (%)		2333 (24)	2265 (23)
Without evidence of cardiovascular or kidney disease, *n* (%)		5161 (53)	5306 (54)
Hypertension registry,^b^ *n* (%)		6022 (61)	6028 (61)
History of smoking, *n* (%)	Current smoker	1258 (13)	1225 (13)
	Past smoker	285 (3)	277 (3)
	Never smoker	4298 (44)	4292 (44)
	Unknown	3983 (41)	4030 (41)
BMI, kg/m^2^, mean (SD), (No. patients with a measurement)		32 (6), (8651)	32 (6), (8445)
HbA1c (%), mean (SD), (No. patients with a measurement)		8 (2), (9774)	8 (2), (9759)
Fasting plasma glucose, mg/dl, mean (SD), (No. patients with a measurement)		174 (61), (9268)	175 (62), (9203)
Low-density lipoprotein, mg/dl, mean (SD), (No. patients with a measurement)		95 (37), (8848)	93.5 (35), (8729)
High-density lipoprotein, mg/dl, mean (SD), (No. patients with a measurement)		43 (11), (9743)	44 (11), (9706)
Systolic BP, mm Hg, mean (SD), (No. patients with a measurement)		133 (15), (9331)	133 (16), (9299)
Diastolic BP, mm Hg, mean (SD), (No. patients with a measurement)		78 (10), (9331)	79 (10), (9299)
**Diabetes medications**			
Metformin, *n* (%)		9211 (94)	9290 (95)
Insulin, *n* (%)	All	1933 (20)	1796 (18)
	Basal insulin	1785 (18)	1694 (17)
	Fast-acting insulin	634 (7)	610 (6)
Sulfonylureas, *n* (%)		1389 (14)	1413 (14)
Glucagon-like peptide-1 receptor agonists, *n* (%)		352 (4)	342 (4)
Thiazolidinediones, *n* (%)		346 (4)	340 (4)
**Cardiovascular medications**			
RAAS inhibitors, *n* (%)		6294 (64)	6237 (64)
Statin, *n* (%)		7613 (78)	7599 (77)
*β* blocker, *n* (%)		3473 (35)	3420 (35)
Antihypertensives, *n* (%)		6965 (71)	6932 (71)
Aldosterone antagonist, *n* (%)		450 (5)	395 (4)
**Kidney markers**			
eGFR, ml/min per 1.73 m^2^, *n* (%)	>90	5573 (57)	5573 (57)
	60–90	3430 (35)	3430 (35)
	45–60	661 (7)	661 (7)
	30–45	160 (2)	160 (2)
	Mean (SD)	89 (18)	90 (19)
UACR, mg/g, *n* (%)	Urine albumin below detectable levels	3589 (37)	3620 (37)
	<15	1467 (15)	1501 (15)
	15–<30	1276 (13)	1296 (13)
	30–<300	2311 (24)	2251 (23)
	≥300	611 (6)	563 (6)
	Missing	570 (6)	593 (6)
	Median (IQR)	12 (0–45)	12 (0–42)

SGLT2i, sodium-glucose transporter 2 inhibitor; DPP4i, dipeptidyl peptidase 4 inhibitors; SES, socioeconomic status; BMI, body mass index; HbA1c, glycated hemoglobin A1c; RAAS, renin-angiotensin-aldosterone system; UACR, urine albumin-to-creatinine ratio; IQR, interquartile range.

a Socioeconomic status was ranked on a 1 (lowest) to 10 (highest) scale. This parameter was categorized into four groups: low (1–3), low-medium (4–5), medium (6–7), and high (8–10).

b Based on Maccabi Healthcare Services's validated registries.

### Main Outcomes

**Figure 1:**
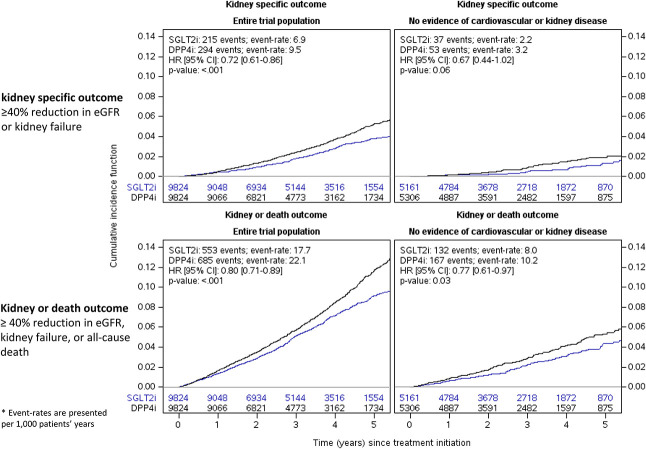
**The association between initiation of sodium-glucose cotransporter 2 inhibitors, compared with dipeptidyl peptidase 4 inhibitors, and the risks of the kidney-specific or kidney-or-death outcomes, in the whole trial population and in patients without evidence of cardiovascular or kidney disease.** The kidney-specific composite outcome included confirmed ≥40% eGFR decline or kidney failure. The kidney-or-death outcome included also all-cause mortality. No evidence of cardiovascular or kidney disease was defined by the lack of the following: evidence of cardiovascular disease, eGFR <60 ml/min per 1.73 m^2^, or urine albumin-to-creatinine ratio ≥30 mg/g. Analysis was performed using the intention-to-treat follow-up. Event rates are presented as events per 1000 patient-years. HR, 95% CI, and *P* values were assessed using Cox proportional hazard regression models. CI, confidence interval; DPP4i, dipeptidyl peptidase 4 inhibitor; HR, hazard ratio; SGLT2i, sodium-glucose transporter 2 inhibitor.

Participants had a median follow-up of 38 months (IQR, 22–55), with an overall duration of 63,145 person-years, and a rate of loss to follow-up of 1% (Supplemental Tables 3 and 4). The composite kidney-specific outcome occurred at an event rate of 6.9 versus 9.5 events per 1000 patient-years with SGLT2i versus DPP4i. The respective event rates of the kidney-or-death outcome were 17.7 versus 22.1. Compared with DPP4is, initiation of SGLT2is was associated with a lower risk for the composite kidney-specific and kidney-or-death outcomes (HR, 0.72; 95% CI, 0.61 to 0.86, and HR, 0.80; 95% CI, 0.72 to 0.89, respectively; *P* < 0.001) (Figure 1). There was no evidence that these associations varied by most baseline subgroups (*P* of interaction ranging from 0.11 to 0.87). The association between initiation of SGLT2is versus DPP4is and the kidney-or-death outcome was more pronounced in patients not treated with angiotensin-converting enzymes/angiotensin II receptor blockers compared with those treated (*P* interaction = 0.04), although reaching separate statistical significance indicated superiority of SGLT2is in each category alone (Supplemental Figure 2). In the subgroup of patients without evidence of cardiovascular or kidney disease, initiation of SGLT2is was associated with a lower risk for the composite kidney-or-death outcome (HR, 0.77; 95% CI, 0.61 to 0.97), but not for the kidney-specific outcome (HR, 0.67; 95% CI, 0.44 to 1.02) (Figure 1 and Supplemental Figure 2). Similar findings were observed in the as-treated analysis (Supplemental Figure 2) or when comparing empagliflozin initiators with DPP4i initiators (Supplemental Tables 5 and 6).

### Other Outcomes

**Figure 2:**
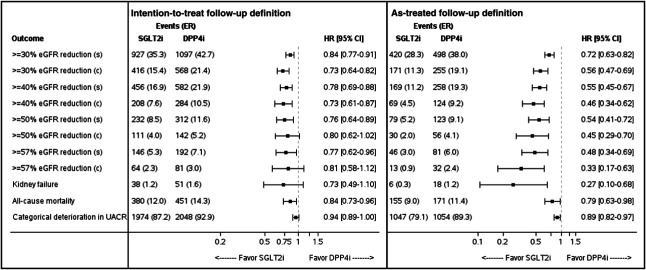
**The association between initiation of sodium-glucose cotransporter 2 inhibitors, compared with dipeptidyl peptidase 4 inhibitors, and other kidney outcomes in the intention-to-treat and as-treated follow-up analyses.** In the intention-to-treat analysis, follow-up continued until the occurrence of an effectiveness outcome (for the specific end point), end of data availability, death, or September 2021. The as-treated analysis's follow-up criteria were those used by the intention-to-treat analysis, added by censoring at study drug discontinuation or the initiation of the comparator drug. In the as-treated analysis, follow-up was extended by a grace period of 90 days after the last drug dispensation. Outcomes are assessed either as single (s) or confirmed (c) measurements. Categorical increase in UACR was defined using the following categories: <30, 30–<300, and ≥300 mg/g. Event rates are presented per 1000 patient-year. HR, 95% CIs, and *P* values were assessed using Cox proportional hazard regression models. ER, event rate; UACR, urine albumin-to-creatinine ratio.

Initiation of SGLT2is versus DPP4is was associated with lower risks of single-measurement ≥30% (HR, 0.84; 95% CI, 0.77 to 0.91), ≥40% (HR, 0.78; 95% CI, 0.69 to 0.88), ≥50% (HR, 0.76; 95% CI, 0.64 to 0.89), and ≥57% (HR, 0.77; 95% CI, 0.62 to 0.96) eGFR loss. Generally, similar findings, although not always reaching statistical significance, were observed for the respective confirmed-measurement eGFR declines (Figure 2). The HRs for a categorical increase in UACR were 0.94 (95% CI, 0.89 to 1.00) and 0.89 (95% CI, 0.82 to 0.97) in the intention-to-treat and as-treated analyses, respectively. Compared with DPP4is, SGLT2i therapy was associated with a lower risk of death from any cause (HR, 0.84; 95% CI, 0.74 to 0.96), but not new kidney failure (38 and 51 patients in SGLT2i and DPP4i groups, respectively; HR, 0.73; 95% CI, 0.49 to 1.10). Generally, similar findings were observed in the as-treated analysis, although reaching statistically significant benefits with SGLT2is versus DPP4is for all tested end points (Figure 2).

### eGFR Slopes

**Figure 3:**
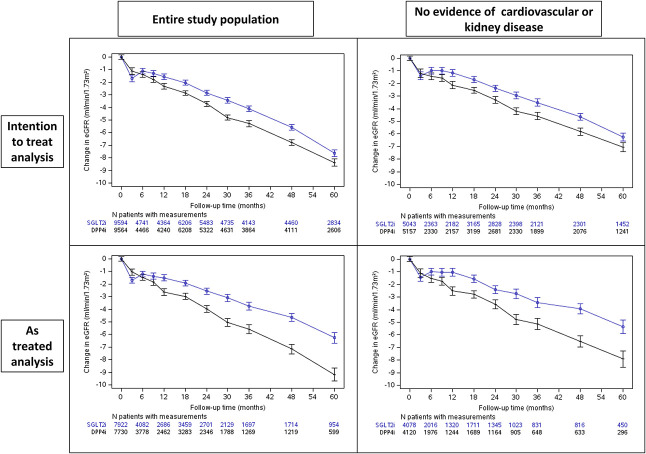
**The association between initiation of sodium-glucose cotransporter 2 inhibitors, compared with dipeptidyl peptidase 4 inhibitors, and the change in eGFR over time.** In the intention-to-treat analysis, follow-up continued until the occurrence of an effectiveness outcome (for the specific end point), end of data availability, death, or September 2021. The as-treated analysis's follow-up criteria were those used by the intention-to-treat analysis, added by censoring at study drug discontinuation or the initiation of the comparator drug. In the as-treated analysis, follow-up was extended by a grace period of 90 days after the last drug dispensation. A subgroup of patients without evidence of cardiovascular or kidney disease was defined by the lack of the following: evidence of cardiovascular disease, eGFR <60 ml/min per 1.73 m^2^, or urine albumin-to-creatinine ratio ≥30 mg/g. Mixed models for repeated measures were used to describe the change in eGFR over time. We defined time windows as follows: every 3 months in the first year, every 6 months between years 1 and 3, and each year thereafter. At each time window, we considered only the eGFR measurement closest to the end of the period for each patient. We calculated the *P* value of the change in eGFR between groups at each time point using a mixed-effect model with repeated measures.

In the SGLT2i group, there was an acute reduction in eGFR followed by stabilization compared with the DPP4i group (Figure 3). Similar findings were observed for the subgroup of patients without evidence of cardiovascular or kidney disease (Figure 3).

**Figure 4:**
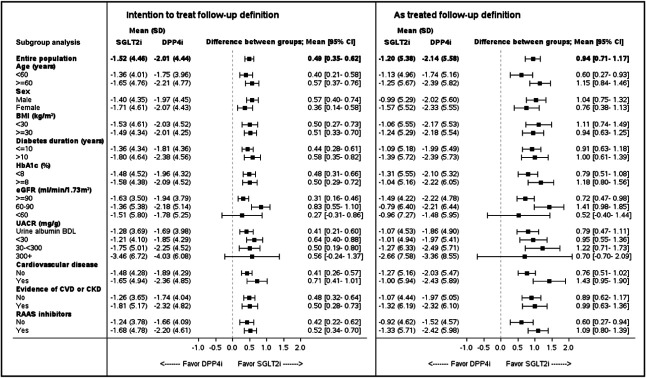
**The association between initiation of sodium-glucose cotransporter 2 inhibitors, compared with dipeptidyl peptidase 4 inhibitors, and annualized eGFR slope overall and by subgroups.** In the intention-to-treat analysis, follow-up continued until the occurrence of an effectiveness outcome (for the specific end point), end of data availability, death, or September 2021. The as-treated analysis's follow-up criteria were those used by the intention-to-treat analysis, added by censoring at-study drug discontinuation or the initiation of the comparator drug. In the as-treated analysis, follow-up was extended by a grace period of 90 days after the last drug dispensation. Without evidence of cardiovascular or kidney disease was defined by the lack of the following: evidence of cardiovascular disease, eGFR <60 ml/min per 1.73 m^2^, or UACR ≥30 mg/g. Mixed models for repeated measures were used to describe the change in eGFR over time. Annualized eGFR slopes were calculated per patient by fitting a linear regression model, followed by calculating the mean eGFR slope for each treatment group, and comparing the groups using a *t*-test. BMI, body mass index; CVD, cardiovascular disease; HbA1c, glycated hemoglobin A1c; RAAS, renin-angiotensin-aldosterone system.

SGLT2i initiation was associated with attenuation of the eGFR slope in both the entire population (between-group difference of 0.49 ml/min per 1.73 m^2^ per year; 95% CI, 0.35 to 0.62) and those without evidence of cardiovascular or kidney disease (0.48 ml/min per 1.73 m^2^ per year; 95% CI, 0.32 to 0.64) (Figure 4). Similar findings were observed in the as-treated analysis, where the between-group differences were 0.94 ml/min per 1.73 m^2^ per year (95% CI, 0.71 to 1.17) and 0.89 ml/min per 1.73 m^2^ per year (95% CI, 0.62 to 1.17) for the overall population and those without evidence of cardiovascular or kidney disease, respectively. These benefits were observed across most other tested subgroups (Figure 4).

## Discussion

Using over 6 years of real-world data and 63,145 person-years, we compared long-term kidney outcomes between patients with type 2 diabetes who initiated SGLT2is versus DPP4is. Patients treated with SGLT2is had a 28% and 20% relative reduction in the risk of the kidney-specific and kidney-or-death outcomes, respectively, compared with DPP4is, along with attenuation of 0.49 ml/min per 1.73 m^2^ per year of eGFR slope. Similar effects were observed also in a subgroup of patients without evidence of cardiovascular or kidney disease.

In a previous study, we showed that in patients with type 2 diabetes, initiation of SGLT2is compared with other glucose-lowering agents is associated with a reduction in kidney risk in a real-world setting.^11^ These trends were observed in subgroups of patients with low kidney risk, although not always reaching statistical significance.^11^ In this analysis, DPP4is were selected as a comparator because of their neutral effects on eGFR loss.^14^ Both classes are often used in a similar disease stage, reducing the risk of bias by indication. Like other studies from Scandinavia,^15^ Hong Kong,^16^ and the United Kingdom^17,18^ that used DPP4is as control, we also found that SGLT2is are associated with better kidney outcomes. This analysis adds several novel aspects. First, compared with previous studies,^8,15,17,18^ we have a relatively long median follow-up (38 months) with a low rate of loss to follow-up, enabling assessment of the long-term effects of the drugs. The between-arm gap of eGFR loss seemed to increase in favor of SGLT2is with longer drug exposure (as-treated analysis). Second, we assessed the risk of an array of commonly used kidney outcomes, including albuminuria progression and eGFR slope over time. Taking advantage of the long-term follow-up, along with comparing eGFR slopes, we were able to find mitigation of eGFR loss, even in patients without evidence of cardiovascular or kidney disease. All in all, these findings indicate that the kidney benefits of SGLT2is are present in various populations treated in the real-world setting.^8,11,16–20^

eGFR slope is increasingly accepted as a surrogate marker for clinical kidney outcomes. It was shown to have more power to detect kidney benefits in populations with high baseline eGFR.^21^ A treatment effect of 0.5–1.0 ml/min per 1.73 m^2^ per year has a strong predictive value for benefits of a composite of ≥57% eGFR loss or kidney failure.^22,23^ We found attenuation of the eGFR slope by 0.49 and 0.94 ml/min per 1.73 m^2^ per year in the intention-to-treat and as-treated analyses, respectively. Similar to recent data from a randomized controlled trial,^24^ SGLT2i therapy was associated with mitigation of eGFR loss in almost all tested subgroups, including those without evidence of cardiovascular or kidney disease.

In cardiovascular outcome trials, SGLT2is improved albuminuria outcomes compared with placebo.^25–28^ However, there is a paucity of data regarding the effect of SGLT2is on albuminuria in real-world settings. A short study (approximately 6 months) that did not use propensity score-matching found a mild reduction in UACR among 273 patients who received dapagliflozin in the real world.^19^ Another study found that the association between initiation of SGLT2is versus DPP4is and risk of new microalbuminuria was lower only in an on-treatment analysis but not in the intention-to-treat analysis.^16^ Similarly, we found a relative reduction (6%–11%) in the risk of categorical albuminuria progression that was statistically significant in the as-treated follow-up and not in the intention-to-treat follow-up. The magnitude of the effect is in line with data from the Empagliflozin Cardiovascular Outcome Event Trial in Type 2 Diabetes Mellitus Patients (EMPA-REG OUTCOME) and Dapagliflozin Effect on Cardiovascular Events - Thrombolysis in Myocardial Infarction 58 (DECLARE-TIMI 58) trials that found a modest 16% and 14% relative reduction in the risk of single-measurement categorical increase in UACR, respectively.^25,27^ The lack of statistically significant effects in the intention-to-treat follow-up can be explained by a dilution of the drug effects, irregular sample collection in real-world settings, high interday variability in UACR levels, and the relatively low kidney risk of our study population. In addition, previous data showed that DPP4is also have modest albuminuria-lowering effects.^14^ Taken together, these analyses fill an important data gap regarding the association between SGLT2i use and albuminuria progression in a real-world setting.

In randomized controlled trials enrolling patients with type 2 diabetes, SGLT2is were shown to improve kidney outcomes compared with placebo. However, these studies included patients with atherosclerotic cardiovascular disease or high cardiovascular risk with limited representation of other populations.^29^ Thus, there are limited data to support the cardiovascular and kidney protective effects of SGLT2is in lower-risk populations. In a recent *post hoc* analysis of the DECLARE-TIMI 58 study, dapagliflozin attenuated eGFR slope across all tested populations, including those without evidence of cardiovascular or kidney disease.^24^ In the current analysis, by combining a large cohort of patients at a relatively low-risk, long-term follow-up, and assessment of eGFR slope, we extend these findings to real-world settings. Taken together, these data suggest that SGLT2is may delay both the onset and progression of kidney disease in patients with type 2 diabetes.

Guidelines recommend using SGLT2is independent of glycemic control in patients with type 2 diabetes and those with kidney disease, with heart failure, or at high risk of cardiovascular disease. There is still a controversy in the field about whether to recommend the use of SGLT2is for kidney protection also in patients with type 2 diabetes and lower cardiovascular risk. This analysis specifically focused on patients without cardiovascular or kidney disease, although some of them were at risk of developing cardiovascular or kidney disease. All in all, our data add to the cumulating findings that support the use of SGLT2is, including empagliflozin, for kidney protection also in patients without evidence of cardiovascular and kidney disease.^17,18,24,25^

This study has several limitations. It is an observational study. It includes one health care maintenance organization, thus requiring external validation from other cohorts. Some of the baseline characteristics significantly varied between the prematched study cohorts. The propensity score formed comparable cohorts by balancing the baseline characteristics, but at the price of a lower representation of the actual real-world population. For example, 28% and 4% of the initiators of SGLT2is were treated at baseline with glucagon-like peptide-1 receptor agonists before and after matching, respectively. Smoking status was part of the propensity score; however, this variable was available in only around 60% of the participants. Most (79%) of the patients in the SGLT2i arm initiated empagliflozin, and the rest initiated dapagliflozin. Other class members, such as canagliflozin, ertugliflozin, and sotagliflozin, were not tested in this analysis, although in cardiovascular and kidney outcome trials, all tested SGLT2is were shown to have similar kidney protective effects.^30^ Finally, unlike in a randomized controlled trial setting, where patients usually continue with the index medication, in the real-world setting, patients may stop the index medication or switch to the comparator. We addressed this limitation by using the intention-to-treat and as-treated follow-up definitions—both showing similar findings.

In conclusion, initiation of treatment with SGLT2is versus DPP4is in a real-world setting was associated with continuous long-term mitigation of eGFR loss in patients with type 2 diabetes, even in those without evidence of cardiovascular or kidney disease at baseline.

## Supplementary Material

## Supplemental Material

Supplemental Methods.

Supplemental Table 1A. Variable definitions on the basis of ICD-9 codes, ATC codes, and Maccabi Healthcare Services's registries.

Supplemental Table 1B. International Classification of Diseases, Ninth Revision (ICD-9) diagnosis codes and current procedural terminology used for defining the cardiovascular disease registry.

Supplemental Table 2. Baseline characteristics of any SGLT2i or DPP4i initiators before propensity score-matching.

Supplemental Table 3. Censoring causes during the trials per each cohort, follow-up definition, and by treatment arm.

Supplemental Table 4. Follow-up durations in the intention-to-treat and as-treated analyses.

Supplemental Table 5. Baseline characteristics before and after propensity score-matching with empagliflozin versus any DPP4i.

Supplemental Table 6. The association between initiation of empagliflozin, compared with any DPP4i, and the risks of the kidney-specific or kidney-or-death outcomes, in the whole trial population.

Supplemental Figure 1. A CONSORT diagram describing the formation of the study population.

Supplemental Figure 2. The association between initiation of SGLT2is, compared with DPP4is, and the risk of (A) the kidney-specific or (B) kidney-or-death outcomes, by subgroups.
