# Supplementary material for: Long-Term, Real-World Kidney Outcomes with SGLT2i versus DPP4i in Type 2 Diabetes without Cardiovascular or Kidney Disease
Source: Clin J Am Soc Nephrol. 2023 Jun 29;18(9):1153–62. doi: 10.2215/CJN.0000000000000218 (PMC10564349; doi:10.2215/CJN.0000000000000218)

Long-Term, Real-World, Kidney Function Changes with SGLT2i versus DPP4i Type 2 Diabetes without Cardiovascular or Kidney Disease

Cheli Melzer-Cohen*[1], Meir Schechter*[2,3,4], Aliza Rozenberg [2,3], Ilan Yanuv [2,3], Dvora R. Sehtman-Shachar[2,3], Alisa Fishkin[2,3], Doron Rosenzweig[5] , Gabriel Chodick [1,6], Avraham Karasik [1,7], Ofri Mosenzon [2,3,**]

* These authors have equally contributed
** Present address: Regeneron Pharmaceutical Inc.

[1] Maccabi Institute for Research and Innovation, Maccabi Healthcare Services, Tel-Aviv, Israel
[2] Diabetes Unit, Department of Endocrinology and Metabolism, Hadassah Medical Center, Jerusalem, Israel
[3] Faculty of Medicine, Hebrew University of Jerusalem, Jerusalem, Israel
[4] Department of Clinical Pharmacy and Pharmacology, University Medical Center Groningen, University of Groningen, Groningen, The Netherlands
[5] Boehringer Ingelheim RCV GmbH and Co. KG, Vienna, Austria
[6] School of Public Health Sackler, Faculty of Medicine, Tel Aviv University, Tel Aviv, Israel
[7] Tel Aviv University, Tel Aviv, Israel

Corresponding author:
Ofri Mosenzon MD MSc
OFRIM@hadassah.org.il
The Diabetes Unit, Department of Endocrinology and Metabolism
Hadassah Ein Kerem Medical Center
P.O.B 12000
Jerusalem 9112001
Israel

Supplemental Methods.
Supplemental Table 1A: Variables definitions based on ICD-9 codes, ATC codes, and Maccabi Healthcare Services' registries.
Supplemental Table 1B: International Classification of Diseases, Ninth Revision [ICD-9] diagnosis codes and Current Procedural Terminology used for defining the cardiovascular disease registry.
Supplemental Table 2: Baseline characteristics of any SGLT2i or any DPP4i initiators before propensity-score matching.
Supplemental Table 3: Censoring causes during the trials per each cohort, follow-up definition, and by treatment arm.
Supplemental Table 4: Follow-up durations in the intention-to-treat and as-treated analyses.
Supplemental Table 5: Baseline characteristics before and after propensity score matching with empagliflozin versus any DPP4i.
Supplemental Table 6: The association between initiation of empagliflozin, compared with any DPP4i, and the risks of the kidney-specific or kidney-or-death outcomes, in the whole trial population.
Supplemental Figure 1: A CONSORT diagram describing the formation of the study population.
Supplemental Figure 2: The association between initiation of SGLT2i, compared with DPP4i, and the risk of (A) the kidney-specific or (B) kidney-or-death outcome, by subgroups.

Supplemental Methods

The following baseline variables were used for the propensity score matching:

Demographics: age, sex, socioeconomic status (1-3, 4-5, 6-7, 8-10), time of entering into study (by year and quartiles), body mass index (BMI; <25, 25-<30, 30-<35, 35-<40, 40+, missing; kg/m^2), smoking status (current smoker, past smoker, never smoker, missing), systolic blood pressure, and diastolic blood pressure.

Co-morbidities: duration (years) of diabetes (<=2, 2-5, 5-10, 10+ years), ischemic heart disease, myocardial infarction, after cardiac revascularization procedure, unstable angina, stable angina, atrial fibrillation, heart failure, cerebrovascular disease, stroke, transient ischemic attack, peripheral vascular disease, bariatric surgery, hypertension, hyperlipidemia, cancer, diabetic neuropathy, diabetic nephropathy, diabetic retinopathy or other ophthalmic manifestations, diabetic foot or lower extremity amputation, erectile dysfunction, acute kidney injury, liver disease, osteoarthritis, obstructive sleep apnea, chronic obstructive pulmonary disease (COPD), hypothyroidism, anxiety, depression, and psychoses.

Medications**:** metformin, sulfonylureas, glucagon-like peptide-1 receptor agonists, thiazolidinediones, meglitinides, alpha-glucosidase inhibitors, long-acting insulin, fast-acting insulin, pre-mix or intermediate insulin, angiotensin-converting-enzyme inhibitor, angiotensin II receptor blocker, antihypertensive drugs, calcium channel blocker, thiazides, loop diuretics, other diuretics, nitrates, other hypertension drugs, statins, PCSK-9 inhibitors, other lipid-lowering drugs (excluding statins), COPD or asthma medications, antiplatelet, anticoagulants, heparin and other low-molecular weight heparins, oral corticosteroids, nonsteroidal anti-inflammatory drug, opioids, antidepressants, antipsychotics, anti-Parkinson agents, mineralocorticoid receptor antagonists, digoxin, agents for dementia, lithium, anti- arrhythmic drugs, bisphosphonates, anti-convulsants, Benzodiazepines, and proton pump inhibitors.

Laboratory examinations: UACR (urine albumin below detectable levels, <15, 15-30, 30-300, 300+, mg/g, or missing), total cholesterol, low-density cholesterol, high-density cholesterol, triglycerides, eGFR, baseline eGFR slope, fasting-plasma glucose, ALT, AST, alkaline phosphatase, BUN, platelets count, and bilirubin.

Definition of history of cardiovascular disease

Evidence of cardiovascular disease was based on the Maccabi Healthcare Systems' (MHS)' registry. Inclusion in this registry requires that patients will be diagnosed at least twice by a hospital or outpatient cardiologist, a primary physician, or a pediatrician, with at least one clinical diagnosis according to the International Classification of Diseases, Ninth Revision [ICD-9] diagnosis coding, or with a relevant Current Procedural Terminology code (see in **Supplementary Table S1B**).

Supplemental Table 1A: Variables definitions based on ICD-9 codes, ATC codes, and Maccabi Healthcare Services' registries.

**Covariate**	**Definition**
Age	Age at treatment initiation
Sex	female, male
Weight	Last evaluation during baseline period
Height	Last evaluation during baseline period
Socioeconomic status	A 1-10 scale of socioeconomic status (1-low, 10-high), including data from Israel Central Bureau of Statistics as well as updates on new settlements.
Blood pressure	Last evaluation during baseline period
Smoking status	Last recorded status during baseline period
**Co-morbidities**	Entered into cardiovascular disease major registry prior to index date
Atherosclerotic cardiovascular disease	Included in MHS cardiovascular disease major registry any time prior to index date
Ischemic heart disease	Included in MHS IHD registry any time prior to index date
Myocardial infarction registry	Included in MHS MI registry any time prior to index date
Myocardial infarction	ICD-9 codes 410.x
PCI (Diagnostic and therapeutic)	ICD-9 codes: 00.66, V45.82, 36.0
CABG	ICD-9 codes: V45.81, 36.1
Cerebrovascular disease	Included in MHS registry any time prior to index date
Stroke	Included in MHS stroke registry any time prior to index date
Transient ischemic attack	Included in MHS TIA registry any time prior to index date
Peripheral vascular disease	Included in MHS PVD registry any time prior to index date or ICD-9 codes: 440.20 – 440.24, 440.29 – 440.32, 440.3, 440.4, 443.9, 38.18, 38.19 , '39.25', '39.29' , '38.08', '38.09', '38.38', '38.39','38.48', '38.49', '39.5X' , '39.9'
Congestive heart disease	Included in MHS CHF registry prior to 1.1.2020
Atrial fibrillation	Included in MHS AF registry prior to 1.1.2020
unstable angina	ICD-9 code: 411.x
Stable angina	ICD-9 code: 413.x
Bariatric surgery	CPT codes: 43644, 43842, 43845, 43848
Hypertension	Included in MHS HTN registry any time prior to index date
Hyperlipidemia	ICD-9 code: 272.0x-272.4x
Cancer	Included in MHS cancer registry any time prior to index date
Erectile dysfunction	ICD-9 codes: 607.84 during baseline period
Diabetes mellitus	Included in MHS diabetes registry prior to 1.1.2020. Diabetes type was based on registry's classification. Among patients without classification, if they were on fast acting insulin, they were considered as type 1. Otherwise, as type 2.
Diabetic neuropathy	ICD-9 codes: 250.6x, 357.2x, 337.1
Diabetic nephropathy	ICD-9 codes: 250.4x, 583.81

Diabetic foot or lower extremity amputation	ICD-9 codes: 707.1x, V49.7x (excluding V49.76 or V49.77)<br>Procedures: 84.10-84.17<br>CPT codes: 27590, 27880, 27884, 28800, 28820
Diabetic retinopathy or other ophthalmic manifestations	ICD-9 codes: 362.0x, 250.5x (without 362.01-362.07), 366.41 (diabetic cataract), 365.44 (diabetic glaucoma), 361.9x, 379.23,<br>Or procedures: 14.7x, 14.24, 14.34, 14.54
Liver disease	ICD-9 codes: 070.x, 570.x- 573.x 456.0x-456.2x, 576.8x, 782.4x, 789.5x, 39.1x, 42.91
kidney failure or on dialysis	Stage 5 CKD based on eGFR evaluation, on dialysis in the past or after kidney transplantation; eGFR<15; ICD-9 CM codes: 585.5x, 585.6x , V56.0x, V56.8x, V45.1x , 39.95, 54.98 , V42.0x, 996.81 , 55.6x or on dialysis
Dialysis	ICD-9 CM code: 39.95, V45.1, V56.0, V56.1; CPT codes: 90935, 90945 or as defined in CKD registry
Acute kidney injury that requires dialysis (ARF-D)	ICD-9 CM code: 39.95, V45.1, V56.0, V56.1; CPT codes: 90935, 90945
Osteoarthritis	ICD-9 codes: 715.x at baseline period
Obstructive Sleep Apnea	ICD-9 codes: 327.23 at baseline period
Hypothyroidism	ICD-9 codes: 244.X at baseline period
COPD	Included in MHS COPD registry any time prior to index date
Anxiety	ICD-9 codes: 293.84, 300.0x, 300.2x, 300.3x, 309.24, 308.0x, 309.81 at baseline period
Depression	ICD-9 codes: 293.83, 296.2x. 296.3x, 298.0x, 300.4x, 309.0x, 309.1x, 309.28, 311.xx at baseline period
Psychoses	ICD-9 codes: 290.8x, 290.9x, 295.xx, 297.xx, 298.xx, 299.xx, 780.1x at baseline period
Gestational ultrasound	CPT codes: 58974, 59000, 76801, 76805, 76810, 76811, 76815, 76816, 76817, 78657, 9 months before index date and during treatment
**Concomitant medications**	
Angiotensin-converting-enzyme inhibitor	≥1 filled dispensation/any other record of the drug use during baseline period. ATC codes: C09A, C09B
Angiotensin II receptor blocker	≥1 filled dispensation/any other record of the drug use during baseline period. ATC codes: C09C, C09D
Beta-blocker	≥1 filled dispensation/any other record of the drug use during baseline period. ATC codes: C07
Calcium channel blocker	≥1 filled dispensation/any other record of the drug use during baseline period. ATC codes: C08, C07FB, C09BB, C09DB
Thiazides	≥1 filled dispensation/any other record of the drug use during baseline period. ATC codes: C03A
Loop diuretics	≥1 filled dispensation/any other record of the drug use during baseline period. ATC codes: C03C
Other diuretics	≥1 filled dispensation/any other record of the drug use during baseline period. ATC codes: C03D
Nitrates	≥1 filled dispensation/any other record of the drug use during baseline period.

	ATC codes: C01DA02, C01DA08, C01DA14,<br>C01EB18
Other hypertension drugs	≥1 filled dispensation/any other record of the drug use during baseline period.<br>ATC codes: C02CA04, C03DA04, C02CA01, G04CA03, C02AC01, C02AC02, C02DB02,<br>C09XA02, C09XA52
PCSK-9 inhibitors	≥1 filled dispensation/any other record of the drug use during baseline period.<br>ATC codes: C10AX13, C10AX14
Statins	≥1 filled dispensation/any other record of the drug use during baseline period.<br>ATC codes: C10AA
Other lipid-lowering drugs, excluding statins	≥1 filled dispensation/any other record of the drug use during baseline period.<br>ATC codes: C10, excluding C10AA, C10BA, C10BX
COPD or asthma medications	≥1 filled dispensation/any other record of the drug use during baseline period.<br>ATC codes: R03AK06,R03AK07, R03AC13, R03AC12, R03AC02, R03CC03, R01AX03, R03BB01, R03BB04, R03DA04, R03DC03, R03DC01, R03BB05, R03AL05, R03AC18, R03AC19, R03BB07, R03AL01, R03AL02, R03AL03, R03AL04, R03AL05, R03AL06, R03AL08, R03AL09
Antiplatelet	≥1 filled dispensation/any other record of the drug use during baseline period.<br>ATC codes: B01AC06 , B01AC04 , B01AC22 , B01AC05 , B01AC07 , B01AC23 , B01AC24
Anticoagulants	≥1 filled dispensation/any other record of the drug use during baseline period.<br>ATC codes: B01AA03 , B01AE07 , B01AF01 , B01AF02
Heparin and other low-molecular weight heparins	≥1 filled dispensation/any other record of the drug use during baseline period.<br>ATC codes: B01AB , B01AE07
Oral corticosteroids	≥1 filled dispensation/any other record of the drug use during baseline period.<br>ATC codes: H02AB10 , H02AB09, H02AB07, H02AB06, H02AB04, H02AB08, H02AB02, H02AB01
NSAIDs	≥1 filled dispensation/any other record of the drug use during baseline period.<br>ATC codes: M01A
Metformin	≥1 filled dispensation/any other record of the drug use during baseline period.<br>ATC codes: A10BA02, A10BD07, A10BD08, A10BD10, A10BD11, A10BD13
Sulfonylureas 2nd generation	≥1 filled dispensation/any other record of the drug use during baseline period.<br>ATC codes: A10BB
Dipeptidyl peptidase-4 inhibitors	≥1 filled dispensation/any other record of the drug use during baseline period.<br>ATC codes: A10BH, A10BD07, A10BD08, A10BD10, A10BD11, A10BD13, A10BD19, A10BD21, A10BD25
Glucagon-like peptide-1 receptor agonists	≥1 filled dispensation/any other record of the drug use during baseline period.<br>ATC codes: A10BJ
Thiazolidinediones	≥1 filled dispensation/any other record of the drug use during baseline period.<br>ATC codes: A10BG
Meglitinides	≥1 filled dispensation/any other record of the drug use during baseline period.<br>ATC codes: A10BX02, A10BX03
Insulin	≥1 filled dispensation/any other record of the drug use during baseline period.<br>ATC codes: A10A

Alpha-glucosidase inhibitors	≥1 filled dispensation/any other record of the drug use during baseline period.<br>ATC codes: A10BF
SGLT-2i	≥1 filled dispensation/any other record of the drug use during baseline period.<br>ATC codes: A10BD15, A10BD19, A10BD20, A10BD21, A10BD25
Opioids	≥1 filled dispensation/any other record of the drug use during baseline period.<br>ATC codes: N02A
Antidepressants	≥1 filled dispensation/any other record of the drug use during baseline period.<br>ATC codes: N06A
Antipsychotics	≥1 filled dispensation/any other record of the drug use during baseline period.<br>ATC codes: N05A
Agents for dementia	≥1 filled dispensation/any other record of the drug use during baseline period.<br>ATC codes: N06D
Antiparkinson agents	≥1 filled dispensation/any other record of the drug use during baseline period.<br>ATC codes: N04
Mineralocorticoid receptor antagonists (MRAs; aldosterone antagonists)	≥1 filled dispensation/any other record of the drug use during baseline period.<br>ATC codes: C03Da
Digoxin	≥1 filled dispensation/any other record of the drug use during baseline period.<br>ATC codes: C01AA05
Neprilysin inhibitors (Valsartan and sacubitril)	≥1 filled dispensation/any other record of the drug use during baseline period.<br>ATC codes: C09DX04
Anti- arrhythmic drugs	≥1 filled dispensation/any other record of the drug use during baseline period.<br>ATC codes: C01B
Lithium	≥1 filled dispensation/any other record of the drug use during baseline period.<br>ATC codes: N05AN
Heparin/LMWH	≥1 filled dispensation/any other record of the drug use during baseline period.<br>ATC codes: B01AB
Bisphosphanates	≥1 filled dispensation/any other record of the drug use during baseline period.<br>ATC codes: M05BA
Anti-convulsant	≥1 filled dispensation/any other record of the drug use during baseline period.<br>ATC codes: N03
Benzodiazepines	≥1 filled dispensation/any other record of the drug use during baseline period.<br>ATC codes: N03AE, N05B, N05C
Proton pump inhibitors	≥1 filled dispensation/any other record of the drug use during baseline period.<br>ATC codes: A02BC
**Laboratory examinations**	
Fasting plasma glucose (mg/dl)	Last measurement during baseline period
HbA1c (%)	Last measurement during baseline period
Total cholesterol (mg/dl)	Last measurement during baseline period
High-density lipoprotein (HDL) level (mg/dl)	Last measurement during baseline period
Low-density lipoprotein (LDL) level (mg/dl)	Last measurement during baseline period

Triglyceride level (mg/dl)	Last measurement during baseline period
Creatinine (mg/dl)	All evaluation in the past 2 years prior to index date. eGFR was calculated from serum creatinine by using CKD-epi equation.
UACR (mg/g)	All evaluations in the past 2 years prior to index date.
Blood urea (mg/dl)	Last measurement in the past 2 years prior to index date.
Liver Function test	Laboratory tests of: ALT, AST, Alkaline Phosphatase, bilirubin total. Last measurement in the past 2 years prior to index date.
Platelets count	Last measurement in the past 2 years prior to index date.

Supplemental Table 1B: International Classification of Diseases, Ninth Revision [ICD-9] diagnosis codes and Current Procedural Terminology used for defining the cardiovascular disease registry.

Condition/procedure	Code(s)	Code type
Coronary artery bypass graft, percutaneous coronary intervention or vascular surgery	33510, 33999, 92980, 92982, 92984, 33999, 33945, 33935, 35141, 35471, 33321, 35081, 37799, 35556, 35565, 75962	CPT
Cerebrovascular accident or transient ischaemic attack	431.x, 433.x-435.x, 438.x, 997.02, 388.02, 342, 442.81, 38.12	ICD-9
Heart failure	398.91, 404.01, 404.03, 404.11, 404.13, 404.91, 404.93, 428.x, 785.51	ICD-9
Ischaemic heart disease	36.x, 411.x, 414.4, 429.1	ICD-9
Myocardial infarction	410.x, 412, 412.9, 429.7, 429.79	ICD-9

Supplemental Table 2: Baseline characteristics of any SGLT2i or any DPP4i initiators before propensity-score matching.

Variable		SGLT2i (n= 16065)	DPP4i (n= 23208)
**Demographics**			
**Age (years);** mean (SD)		61.5 (10.4)	62.5 (12.3)
**Women (%);** n (%)		6046 (37.6)	10173 (43.8)
**Socioeconomic status*;** n (%)	1-3	2165 (13.5)	2656 (11.4)
	4-5	4705 (29.3)	6805 (29.3)
	6-7	5449 (33.9)	8425 (36.3)
	8-10	3721 (23.2)	5289 (22.8)
	Missing	25 (0.2)	33 (0.1)
**Year of study entry;** n (%)	2015-2016	3895 (24.2)	8966 (38.7)
	2017-2018	5684 (35.4)	7670 (33.1)
	2019-2020	6486 (40.4)	6572 (28.3)
**Medical history**			
**Years in diabetes registry;** mean (SD)		9.7 (6.3)	7.4 (5.8)
**Established cardiovascular disease history**;** n (%)		4975 (31.0)	4343 (18.7)
**Without evidence of cardiovascular or kidney disease;** n (%)		7340 (45.7)	13081 (56.4)
**Hypertension registry**;** n (%)		10545 (65.6)	14359 (61.9)
**History of smoking;** n (%)	Current smoker	1989 (12.4)	2878 (12.4)
	Past smoker	517 (3.2)	718 (3.1)
	Never smoker	7407 (46.1)	10110 (43.6)
	Unknown	6152 (38.3)	9502 (40.9)
**BMI kg/m²;** mean (SD), [number of patients with a measurement]		32.5 (5.6), [14448]	31.1 (5.7), [20036]
**HbA1c (%);** mean (SD), [number of patients with a measurement]		8.2 (1.6), [16002]	8.1 (1.7), [22987]
**Fasting plasma glucose (mg/dL);** mean (SD), [number of patients with a measurement]		172.9 (59.8), [15251]	175.5 (62.6), [21773]
**Low-density lipoprotein (mg/dL);** mean (SD), [number of patients with a measurement]		88.9 (35.6), [14593]	98.4 (37.0), [20796]
**High--density lipoprotein (mg/dL);** mean (SD), [number of patients with a measurement]		42.5 (10.4), [15951]	44.4 (11.3), [22906]

**Systolic blood pressure (mmHg);** mean (SD), [number of patients with a measurement]		133.3 (15.5), [15403]	132.7 (15.6), [21954]
**Diastolic blood pressure (mmHg);** mean (SD), [number of patients with a measurement**]**		77.8 (9.6), [15403]	77.9 (9.7), [21954]
**Medications**			
**Diabetes medications**			
**Metformin;** n (%)		14940 (93.0)	22009 (94.8)
**Insulin;** n (%)	All	5573 (34.7)	2791 (12.0)
	Basal insulin	5238 (32.6)	2599 (11.2)
	Fast-acting insulin	1956 (12.2)	829 (3.6)
**Sulfonylureas;** n (%)		2732 (17.0)	3159 (13.6)
**Glucagon-like peptide-1 receptor agonists;** n (%)		4435 (27.6)	350 (1.5)
**Thiazolidinediones;** n (%)		1102 (6.9)	504 (2.2)
**Cardiovascular medications**			
**RAAS inhibitors;** n (%)		11459 (71.3)	13973 (60.2)
**Statin;** n (%)		13242 (82.4)	17356 (74.8)
**Beta blocker;** n (%)		6743 (42.0)	7546 (32.5)
**Antihypertensives;** n (%)		12422 (77.3)	15796 (68.1)
**Aldosterone antagonist;** n (%)		989 (6.2)	804 (3.5)
**kidney markers**			
**eGFR (ml/min/1.73m²);** n (%)	>90	8887 (55.3)	12291 (53.0)
	60-90	5838 (36.3)	7910 (34.1)
	45-60	1113 (6.9)	1822 (7.9)
	30-45	227 (1.4)	1185 (5.1)
	Mean (SD)	89.0 (18.1)	87.0 (20.8)
**UACR (mg/g);** n (%)	Below detectable	5510 (34.3)	9079 (39.1)
	<15	2303 (14.3)	3075 (13.2)
	15-<30	2115 (13.2)	2849 (12.3)
	30-<300	4087 (25.4)	5107 (22.0)
	>=300	1247 (7.8)	1266 (5.5)
	Missing	803 (5.0)	1832 (7.9)
	Median (IQR)	14.2 (0.0- 55.8)	10.4 (0.0- 40.2)

* Socioeconomic status was ranked on a 1 (lowest) to 10 (highest) scale. This parameter was categorized into 4 groups (low [1-3], low-medium [4-5], medium [6-7], and high [8-10]).
**Based on Maccabi Healthcare Services validated registries.

Abbreviations: BMI **–** body mass index; DPP4i - Dipeptidyl peptidase 4 inhibitors; eGFR – estimated glomerular filtration rate; GLP-1 RA – glucagon-like peptide 1 receptor agonist; RAAS – renin

angiotensin aldosterone system; SGLT2i – sodium-glucose transporter 2 inhibitors; UACR **–** urine albumin-to-creatinine ratio.

Supplemental Table 3: Censoring causes during the trials per each cohort, follow-up definition, and by treatment arm.

Cohort		End of study September 30, 2021, n (%)	Lost to follow-up, n (%)	Treatment discontinuation/ switch, n (%)	All-cause mortality, n (%)
**Intention to treat**	**SGLT2i**	9335 (95.0)	109 (1.1)	NA	380 (3.9)
	**DPP4i**	9292 (94.6)	81 (0.8)	NA	451 (4.6)
	**Total**	18627 (94.8)	190 (1.0)	NA	831 (4.2)
**As treated**	**SGLT2i**	4003 (40.7)	47 (0.5)	5619 (57.2)	155 (1.6)
	**DPP4i**	2795 (28.5)	38 (0.4)	6820 (69.4)	171 (1.7)
	**Total**	6798 (34.6)	85 (0.4)	12439 (63.3)	326 (1.7)
**Intention to treat**	**Empagliflozin**	7614 (95.2)	80 (1.0)	NA	302 (3.8)
	**DPP4i**	7517 (94.0)	77 (1.0)	NA	402 (5.0)
	**Total**	15131 (94.6)	157 (1.0)	NA	704 (4.4)
**As treated**	**Empagliflozin**	3440 (43.0)	35 (0.4)	4394 (55.0)	127 (1.6)
	**DPP4i**	2343 (29.3)	37 (0.5)	5445 (68.1)	171 (2.1)
	**Total**	5783 (36.2)	72 (0.5)	9839 (61.5)	298 (1.9)

Abbreviations: DPP4i - Dipeptidyl peptidase 4 inhibitors; SGLT2i – sodium-glucose transporter 2 inhibitors.

Supplemental Table 4: Follow-up durations in the intention-to-treat and as-treated analyses.

	Intention-to-treat			As-treated		
	**SGLT2i**	**DPP4i**	**Total**	**SGLT2i**	**DPP4i**	**Total**
**Mean months (SD)**	39.2 (19.3)	39.0 (20.1)	39.1 (19.7)	21.3 (18.9)	18.7 (16.9)	20.0 (18.0)
**Median months [IQR]**	39.4 [21.9-54.9]	36.3 [21.9-55.0]	37.9 [21.9-55.0]	14.1 [6.0-31.7]	12.7 [5.5-26.7]	13.4 [5.8-28.9]
**Total person-years**	31647	31498	63145	17159	15066	33225

Supplemental Table 5: Baseline characteristics before and after propensity score matching with empagliflozin versus any DPP4i.

		Before matching		After matching	
**Variable**		**Empagliflozin (n= 12434)**	**DPP4i (n= 19269)**	**Empagliflozin (n= 7996)**	**DPP4i (n= 7996)**
**Demographics**					
**Age (years);** mean (SD)		61.9 (10.4)	62.5 (12.3)	61.6 (10.9)	61.6 (12.2)
**Women (%);** n (%)		4426 (35.6)	8477 (44.0)	3025 (37.8)	3055 (38.2)
**Socioeconomic status*;** n (%)	1-3	1526 (12.3)	2193 (11.4)	912 (11.4)	926 (11.6)
	4-5	3661 (29.4)	5686 (29.5)	2368 (29.6)	2316 (29.0)
	6-7	4268 (34.3)	7009 (36.4)	2818 (35.2)	2825 (35.3)
	8-10	2957 (23.8)	4352 (22.6)	1885 (23.6)	1914 (23.9)
	Missing	22 (0.2)	29 (0.2)	13 (0.2)	15 (0.2)
**Year of study entry;** n (%)	2015-2016	2289 (18.4)	5027 (26.1)	1334 (16.7)	1434 (18.0)
	2017-2018	4761 (38.3)	7670 (39.8)	3149 (39.4)	2887 (36.1)
	2019-2020	5384 (43.3)	6572 (34.1)	3513 (43.9)	3675 (45.9)
**Medical history**					
**Years in diabetes registry;** mean (SD)		9.6 (6.3)	7.4 (5.9)	8.0 (6.0)	7.9 (5.9)
**Established cardiovascular disease history**;** n (%)		4268 (34.3)	3479 (18.1)	2034 (25.4)	2038 (25.5)
**Without evidence of cardiovascular or kidney disease;** n (%)		5331 (42.9)	10898 (56.6)	4037 (50.5)	4133 (51.7)
**Hypertension registry**;** n (%)		8179 (65.8)	11778 (61.1)	4923 (61.6)	4958 (62.0)
**History of smoking;** n (%)	Current smoker	1563 (12.6)	2275 (11.8)	1023 (12.8)	1002 (12.5)
	Past smoker	376 (3.0)	516 (2.7)	223 (2.8)	219 (2.7)
	Never smoker	5489 (44.1)	7682 (39.9)	3336 (41.7)	3346 (41.8)
	Unknown	5006 (40.3)	8796 (45.6)	3414 (42.7)	3429 (42.9)

**BMI kg/m²;** mean (SD), [number of patients with a measurement]		32.3 (5.6), [11101]	31.1 (5.7), [16405]	31.7 (5.6), [6990]	31.9 (6.0), [6847]
**HbA1c (%);** mean (SD), [number of patients with a measurement]		8.1 (1.6), [12387]	8.0 (1.7), [19088]	8.0 (1.6), [7956]	8.0 (1.7), [7938]
**Fasting plasma glucose (mg/dL);** mean (SD), [number of patients with a measurement]		172.6 (59.7), [11777]	176.1 (62.9), [18009]	173.9 (60.3), [7516]	173.8 (60.7), [7465]
**Low-density lipoprotein (mg/dL);** mean (SD), [number of patients with a measurement]		88.1 (35.6), [11262]	98.3 (36.6), [17238]	94.2 (36.3), [7194]	92.9 (35.5), [7118]
**High--density lipoprotein (mg/dL);** mean (SD), [number of patients with a measurement]		42.4 (10.3), [12340]	44.4 (11.0), [19006]	43.4 (10.5), [7922]	43.5 (10.8), [7895]
**Systolic blood pressure (mmHg);** mean (SD), [number of patients with a measurement]		133.4 (15.5), [11908]	132.8 (15.7), [18176]	133.2 (15.4), [7578]	133.3 (15.7), [7572]
**Diastolic blood pressure (mmHg);** mean (SD), [number of patients with a measurement]		77.8 (9.6), [11908]	78.0 (9.7), [18176]	78.3 (9.7), [7578]	78.3 (9.8), [7572]
**Medications**					
**Diabetes medications**					
**Metformin;** n (%)		11619 (93.4)	18287 (94.9)	7487 (93.6)	7541 (94.3)
**Insulin;** n (%)	All	4036 (32.5)	2183 (11.3)	1483 (18.5)	1403 (17.5)
	Basal insulin	3802 (30.6)	2044 (10.6)	1378 (17.2)	1324 (16.6)
	Fast-acting insulin	1415 (11.4)	636 (3.3)	512 (6.4)	480 (6.0)
**Sulfonylureas;** n (%)		1955 (15.7)	2361 (12.3)	1028 (12.9)	1026 (12.8)
**GLP-1 RA;** n (%)		3078 (24.8)	246 (1.3)	258 (3.2)	245 (3.1)
**Thiazolidinediones;** n (%)		738 (5.9)	396 (2.1)	272 (3.4)	273 (3.4)

**Cardiovascular medications**					
**RAAS inhibitors;** n (%)		8916 (71.7)	11457 (59.5)	5148 (64.4)	5114 (64.0)
**Statin;** n (%)		10313 (82.9)	14269 (74.1)	6236 (78.0)	6231 (77.9)
**Beta blocker;** n (%)		5415 (43.5)	6159 (32.0)	2918 (36.5)	2912 (36.4)
**Antihypertensives;** n (%)		9688 (77.9)	12971 (67.3)	5713 (71.4)	5719 (71.5)
**Aldosterone antagonist;** n (%)		819 (6.6)	662 (3.4)	369 (4.6)	374 (4.7)
**kidney markers**					
**eGFR (ml/min/1.73m^2);** n (%)	>90	6660 (53.6)	10239 (53.1)	4404 (55.1)	4404 (55.1)
	60-90	4624 (37.2)	6545 (34.0)	2853 (35.7)	2853 (35.7)
	45-60	958 (7.7)	1528 (7.9)	599 (7.5)	599 (7.5)
	30-45	192 (1.5)	957 (5.0)	140 (1.8)	140 (1.8)
	Mean (SD)	88.2 (18.3)	87.1 (20.8)	88.7 (18.4)	89.2 (19.1)
**UACR (mg/g);** n (%)	Below detectable	4190 (33.7)	7354 (38.2)	2864 (35.8)	2913 (36.4)
	<15	1797 (14.5)	2637 (13.7)	1191 (14.9)	1212 (15.2)
	15-<30	1637 (13.2)	2421 (12.6)	1048 (13.1)	1033 (12.9)
	30-<300	3195 (25.7)	4303 (22.3)	1921 (24.0)	1896 (23.7)
	>=300	1003 (8.1)	1052 (5.5)	510 (6.4)	478 (6.0)
	Missing	612 (4.9)	1502 (7.8)	462 (5.8)	464 (5.8)
	Median (IQR)	14.5 (0.0- 58.5)	10.8 (0.0- 40.9)	12.2 (0.0- 46.8)	12.0 (0.0- 43.8)

* Socioeconomic status was ranked on a 1 (lowest) to 10 (highest) scale. This parameter was categorized into 4 groups (low [1-3], low-medium [4-5], medium [6-7], and high [8-10]).

**Based on Maccabi Healthcare Services validated registries.

Abbreviations: BMI – body mass index; DPP4i - Dipeptidyl peptidase 4 inhibitors; eGFR – estimated glomerular filtration rate; GLP-1 RA – glucagon-like peptide 1 receptor agonist; RAAS – renin angiotensin aldosterone system; SGLT2i – sodium-glucose transporter 2 inhibitors; UACR – urine albumin-to-creatinine ratio.

Supplemental Table 6: The association between initiation of empagliflozin, compared with any DPP4i, and the risks of the kidney-specific or kidney-or-death outcomes, in the whole trial population.

	**Follow-up definition**	**Empagliflozin events (ER)**	**DPP4i Events (ER)**	**HR (95%CI)**	**P-Value**
**Kidney specific**	Intention-to-treat	175 (7.3)	218 (9.1)	0.79 (0.65-0.97)	0.021
	As-treated	65 (4.7)	98 (8.2)	0.55 (0.40-0.76)	<0.001
**Kidney or Death**	Intention-to-treat	439 (18.3)	575 (24.1)	0.76 (0.67-0.85)	<0.001
	As-treated	182 (13.3)	255 (21.4)	0.61 (0.51-0.73)	<0.001

The kidney-specific composite outcome included sustained ≥40% eGFR decline or kidney failure. The kidney-or-death outcome included also all-cause mortality. Event rates are presented per 1000 patient-year. Hazard ratios (HR), 95% confidence intervals, and p-values were assessed using Cox proportional hazards regression models.

Abbreviations: DPP4i – Dipeptidyl peptidase 4 inhibitors; eGFR – estimated glomerular filtration rate; ER – event rate; UACR – urine albumin-to-creatinine ratio.

Supplemental Figures

Supplemental Figure 1: A CONSORT diagram describing the formation of the study population.

Abbreviations: DPP4i – Dipeptidyl peptidase 4 inhibitors; eGFR – estimated glomerular filtration rate; MHS - Maccabi Healthcare Services; SGLT2i – sodium-glucose transporter 2 inhibitors.

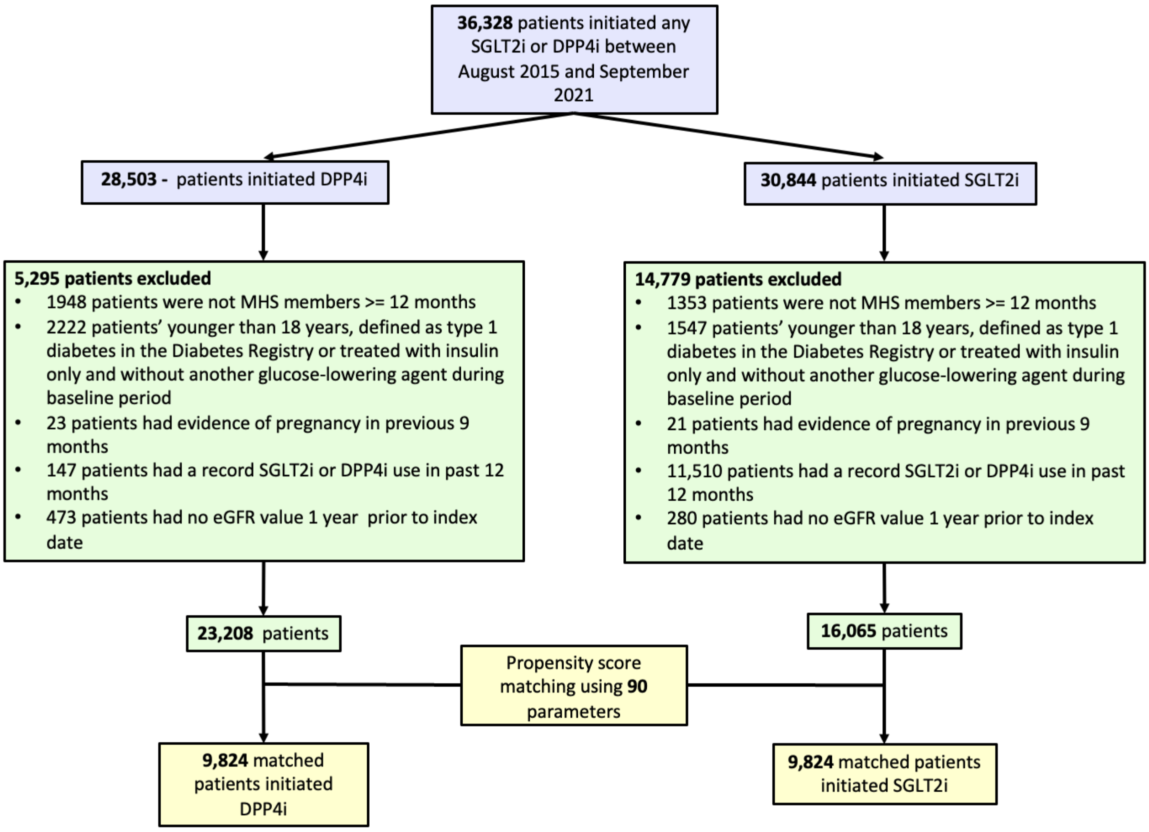

Supplemental Figure 2: The association between initiation of SGLT2i, compared with DPP4i, and the risk of (A) the kidney-specific or (B) kidney-or-death outcome, by subgroups.

The kidney-specific composite outcome included sustained ≥40% eGFR decline or kidney failure. The kidney-or-death outcome included also all-cause mortality. Without evidence of cardiovascular or kidney disease was defined by lack of the following: evidence of cardiovascular disease, eGFR<60 mL/min/1.73 m^2, or UACR≥30 mg/g. Hazard ratios (HR), 95% confidence intervals, and p-values were assessed using Cox proportional hazards regression models.

Abbreviations: BDL – below detectable levels; BMI – body mass index; CVD – cardiovascular disease; CKD – chronic kidney disease; DPP4i - dipeptidyl peptidase 4 inhibitors; eGFR – estimated glomerular filtration rate; HbA1c – glycated hemoglobin A1c; RAAS – renin angiotensin aldosterone system; SGLT2i – sodium-glucose transporter 2 inhibitors; UACR **–** urine albumin-to-creatinine ratio.

A – Kidney-specific outcome

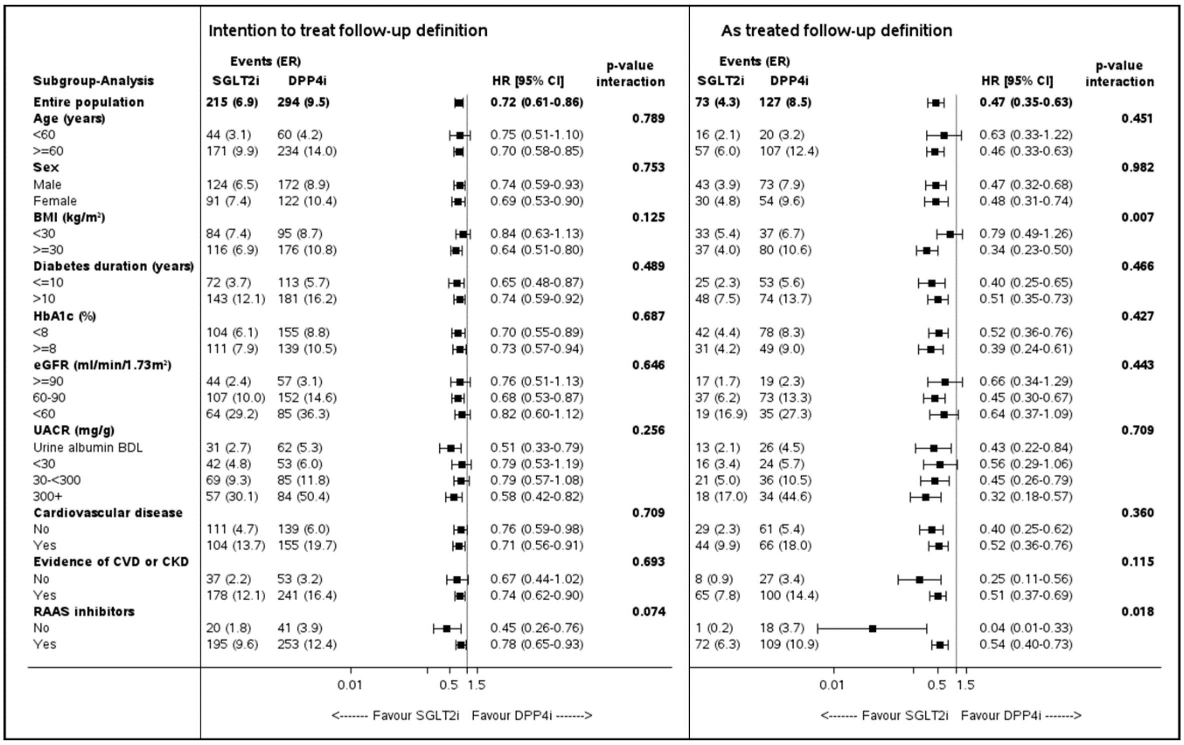

B – Kidney-or-death outcome

Subgroup-Analysis	Intention to treat follow-up definition: Events (ER) SGLT2i	Events (ER) DPP4i	HR [95% CI]	p-value interaction	As treated follow-up definition: Events (ER) SGLT2i	Events (ER) DPP4i	HR [95% CI]	p-value interaction
**Entire population**	**553 (17.7)**	**685 (22.1)**	**0.80 (0.71-0.89)**		**219 (12.8)**	**288 (19.3)**	**0.64 (0.54-0.77)**	
**Age (years)**				**0.865**				0.834
<60	96 (6.8)	122 (8.5)	0.80 (0.62-1.05)		38 (5.0)	44 (7.0)	0.69 (0.45-1.07)	
>=60	457 (26.5)	563 (33.7)	0.78 (0.69-0.88)		181 (19.0)	244 (28.2)	0.66 (0.54-0.80)	
**Sex**				**0.816**				0.436
Male	348 (18.3)	439 (22.8)	0.81 (0.70-0.93)		146 (13.4)	178 (19.2)	0.68 (0.54-0.84)	
Female	205 (16.7)	246 (20.9)	0.79 (0.66-0.94)		73 (11.8)	110 (19.5)	0.59 (0.44-0.79)	
**BMI (kg/m²)**				**0.351**				0.111
<30	239 (21.0)	276 (25.2)	0.83 (0.70-0.99)		101 (16.5)	117 (21.2)	0.78 (0.59-1.01)	
>=30	263 (15.7)	342 (20.9)	0.75 (0.64-0.87)		102 (11.0)	139 (18.5)	0.56 (0.43-0.73)	
**Diabetes duration (years)**				**0.844**				0.819
<=10	218 (11.2)	279 (14.1)	0.80 (0.67-0.95)		88 (8.2)	124 (13.0)	0.62 (0.47-0.82)	
>10	335 (28.4)	406 (36.2)	0.77 (0.67-0.89)		131 (20.4)	164 (30.4)	0.65 (0.52-0.82)	
**HbA1c (%)**				**0.239**				0.679
<8	259 (15.1)	362 (20.6)	0.74 (0.63-0.86)		110 (11.5)	175 (18.6)	0.61 (0.48-0.78)	
>=8	291 (20.8)	323 (24.5)	0.84 (0.72-0.98)		108 (14.5)	113 (20.7)	0.65 (0.49-0.85)	
**eGFR (ml/min/1.73m²)**				**0.911**				0.643
>=90	150 (8.1)	181 (9.9)	0.82 (0.66-1.02)		61 (6.1)	70 (8.6)	0.69 (0.49-0.97)	
60-90	267 (25.0)	315 (30.2)	0.82 (0.70-0.96)		104 (17.4)	141 (25.8)	0.67 (0.52-0.86)	
<60	136 (62.1)	189 (80.8)	0.78 (0.63-0.96)		54 (47.9)	77 (60.0)	0.81 (0.57-1.14)	
**UACR (mg/g)**				**0.110**				0.255
Urine albumin BDL	116 (9.9)	164 (14.1)	0.71 (0.56-0.90)		44 (7.0)	79 (13.8)	0.50 (0.35-0.73)	
<30	115 (13.3)	142 (16.2)	0.82 (0.64-1.04)		47 (9.9)	58 (13.7)	0.69 (0.47-1.01)	
30-<300	181 (24.4)	193 (26.8)	0.91 (0.74-1.10)		72 (17.2)	76 (22.2)	0.75 (0.54-1.05)	
300+	98 (51.8)	137 (82.2)	0.62 (0.48-0.80)		38 (35.8)	52 (68.2)	0.47 (0.31-0.72)	
**Cardiovascular disease**				**0.843**				0.334
No	284 (12.0)	333 (14.4)	0.82 (0.70-0.96)		96 (7.6)	144 (12.8)	0.58 (0.45-0.75)	
Yes	269 (35.4)	352 (44.7)	0.80 (0.69-0.94)		123 (27.6)	144 (39.3)	0.69 (0.54-0.87)	
**Evidence of CVD or CKD**				**0.681**				0.165
No	132 (8.0)	167 (10.2)	0.77 (0.61-0.97)		45 (5.1)	80 (10.0)	0.51 (0.35-0.73)	
Yes	421 (28.6)	518 (35.3)	0.81 (0.72-0.92)		174 (20.8)	208 (30.0)	0.67 (0.55-0.82)	
**RAAS inhibitors**				**0.040**				0.005
No	89 (8.1)	136 (12.8)	0.62 (0.48-0.81)		25 (4.5)	61 (12.4)	0.35 (0.22-0.56)	
Yes	464 (22.9)	549 (27.0)	0.85 (0.75-0.96)		194 (16.9)	227 (22.7)	0.72 (0.60-0.88)	

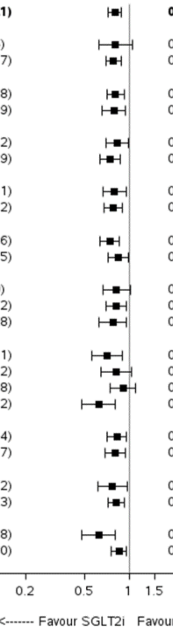

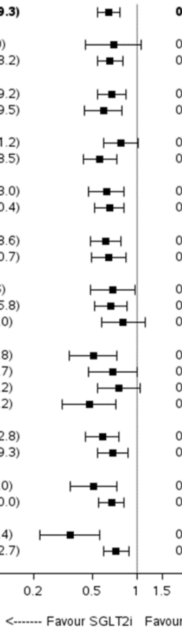

Supplement: Supplementary file 1 [file cjasn-18-1153-s001.pdf]
